# Efficacy of supplemental oxygen in reducing the need for laser or intravitreal bevacizumab in preterm infants with stage 2 retinopathy of prematurity

**DOI:** 10.1186/s12886-024-03483-w

**Published:** 2024-05-24

**Authors:** Robert Minturn, Kelly Hartigan, Sravanthi Vegunta, Charline Boente, Lilian Golzarri-Arroyo, Elizabeth Hynes, Elleen Laughlin, Kathryn Haider, Kok Lim Kua

**Affiliations:** 1 Indiana University School of Medicine, Indianapolis, IN USA; 2 Department of Ophthalmology, Indiana University School of Medicine, Indianapolis, IN USA; 3 Department of Epidemiology and Biostatistics, Indiana University School of Public Health Bloomington, Bloomington, IN USA; 4 Herman B Wells Center for Pediatric Research, Indianapolis, IN USA; 5 Department of Pediatrics, Division of Neonatal- Perinatal Medicine, Indiana University School of Medicine, Indianapolis, IN USA

**Keywords:** Retinopathy of prematurity, Supplemental oxygen, Neonatal outcomes, Visual loss

## Abstract

**Background:**

Retinopathy of prematurity (ROP) is a disease that affects preterm infants born younger than 30 weeks of gestation. The pathophysiology of ROP involves an initial vaso-obliterative phase followed by vaso-proliferative phase that leads to disease progression. The use of supplemental oxygen during the vaso-proliferative phase of ROP has been associated with reduced disease progression, but how this impacts the need for ROP treatment is unclear. The goal of this study was to compare the rate of laser or intravitreal bevacizumab after implementation of a new supplemental oxygen therapy protocol in preterm infants with stage 2 ROP.

**Methods:**

This is a retrospective chart review of preterm infants diagnosed with stage 2 ROP at Riley Hospital for Children between 1/2017 and 12/2022. Patients diagnosed between 1/2017 and 6/2020 were classified as Cohort A, preprotocol implementation. Patients diagnosed from 8/2020 to 12/2022 were classified as Cohort B, postprotocol implementation. In Cohort A, oxygen saturation was kept at 91-95% through the entire hospitalization. In Cohort B, oxygen saturation was increased to 97–99% as soon as Stage 2 ROP was diagnosed. Statistical analyses were performed using chi-square and Student’s T test, followed by multivariate analyses to determine the impact of the oxygen protocol on the need for ROP treatment.

**Results:**

A total of 211 patients were diagnosed with stage 2 ROP between 1/2017 and 12/2022. Of those patients, 122 were before protocol implementation therapy (Cohort A), and 89 were after implementation of supplemental oxygen protocol (Cohort B). Gestational age was slightly higher in Cohort B (Cohort A 25.3 ± 1.9, Cohort B 25.8 ± 1.84, *p* = 0.04). There was no difference in birth weight, NEC, BPD, or survival. Cohort B had lesser need for invasive mechanical ventilation and higher days on CPAP during hospitalization. Notably, Cohort A had 67 (55%) patients treated with laser photocoagulation or intravitreal bevacizumab versus 20 (22%) patients in Cohort B (OR 0.19, 0.08–0.40).

**Conclusion:**

The need for laser photocoagulation or intravitreal bevacizumab was significantly decreased in high-risk patients treated with the supplemental oxygen protocol. This result supports the idea that targeted supplemental oxygen therapy to keep saturations between 97 and 99% can reduce disease progression in infants with stage 2 ROP and potentially decrease the burden of additional procedures.

## Introduction

Retinopathy of prematurity (ROP) is a disease that primarily affects premature infants born before 30 weeks of gestation and is currently the leading cause of childhood blindness in the United States [1]. The pathophysiology of ROP consists of two phases: an initial vaso-obliterative phase followed by a vaso-proliferative phase. The initial vaso-obliterative phase is due to the transition from a hypoxic in-utero environment to a relatively hyperoxic ex-utero environment at birth. This phase usually occurs before the corrected gestation age (CGA) of 30 weeks, when the use of oxygen (O2) and higher target O2 saturations result in increased ROP severity [2]. The vaso-proliferative phase occurs after 30–32 weeks of CGA, and the pathophysiology is complex, involving a combination of factors including retinal hypoxia leading to increased angiogenic factors such as vascular endothelial growth factor (VEGF), as well as other nutrient regulated factors such as insulin-like growth factor 1 (IGF-1) [3, 4]. 

Current management of ROP involves limiting O2 during the first few weeks of life and serial ophthalmology retinal examinations beginning at 30 weeks CGA or later. ROP is classified based on zone, stage, and type as defined by ICROP criteria [5]. ROP deemed high risk for disease progression that could result in a poor visual or anatomic outcome (Type 1 ROP) is treated with laser photocoagulation and/or intravitreal anti-VEGF antibody (bevacizumab) [6]. Several studies have reported an association between laser photocoagulation and decreased visual acuity and a higher rate of myopia and astigmatism during childhood [7, 8]. Infants received intravitreal bevacizumab treatment may experience reactivation of ROP [9]. Intravitreal bevacizumab has also been reported to be associated with a higher risk of severe neurodevelopmental impairment and cerebral palsy [10, 11]. It is essential to note that these findings may be influenced by biases, as the associated morbidities may be partly attributed to the illness severity of the premature infants [7]. Therefore, there is a pressing need to explore alternative, less invasive approaches that can impede ROP progression and potentially avoid the need for laser or intravitreal bevacizumab.

Most of the efforts to prevent ROP are focused on limiting O2 delivery during the vaso-obliterative phase [12, 13]. Large, randomized control trials in the US, Australia, New Zealand, and United Kingdom found that lower oxygen saturations (85–89%) were associated with decreased rates of ROP in preterm infants compared to 91–95% target saturations [2, 14]. These studies randomized premature infants shortly after birth up until 36 CGA and therefore likely assessed the benefits of lower oxygen exposure during the vaso-obliterative phase, but unfortunately found a higher rate of mortality and necrotizing enterocolitis (NEC). Additional retrospective cohort studies also observed that infants managed with biphasic oxygen saturation targets (85–92% before, 92–97% after 34 weeks corrected gestational age), as opposed to static (91–95% throughout hospital stay) had lower ROP severity [12, 13]. To date, only one randomized control trial assessed oxygen supplementation in preventing ROP progression (STOP-ROP) after established ROP [15]. This study did not identify statistically significant benefits or harm of O2 supplementation on ROP. However, post hoc analysis suggested the benefits of O2 supplementation in prethreshold ROP without plus disease. To the best of our knowledge, small retrospective studies have also suggested potential benefits of supplemental O2 in preventing ROP progression [16, 17], but none have shown the direct impact of O2 supplementation after 32 weeks of CGA (during the vaso-proliferative phase) on the need for laser or intravitreal bevacizumab.

Taking these background studies into consideration, we implemented a protocol using supplemental O2 to prevent ROP progression in preterm infants with stage 2 ROP. We hypothesize that optimizing supplemental oxygen therapy in patients with stage 2 ROP can decrease the risk of progression to type I ROP and therefore reduce the need for treatment with either laser photocoagulation or intravitreal bevacizumab.

## Methods

### Study design

**Fig. 1:**
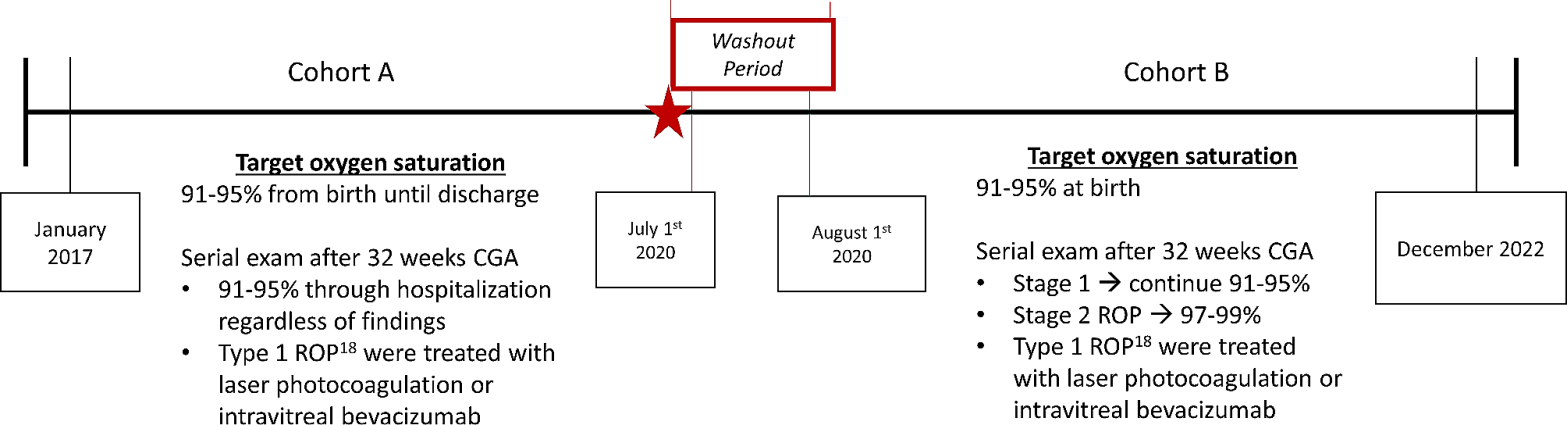
Study timeline from jan 2017 to dec 2022 and brief description of differences in target oxygen saturations. The implementation denoted by the star followed by 1 month washout period after implementation

This is a retrospective chart review of neonates discharged from the neonatal ICU (NICU) at the Riley Hospital for Children between 1/1/2017 and 12/31/2022. Exemptions were obtained from the Indiana University School of Medicine (IUSM) Institutional Review Board (IRB), and the need for informed consent was waived because only deidentified clinical data was analyzed for this study. All data were deidentified and in compliance with the Healthcare Insurance Portability and Accountability Act (HIPAA). The new supplemental oxygen protocol was implemented in July 2019. We defined our cohorts as preprotocol implementation (Cohort A), which spanned from 1/2017 to 6/2020, and postprotocol implementation (Cohort B), which spanned 8/2019-12/2022 (Fig. 1). Preterm infants diagnosed in July 2020 were excluded to provide a washout period, thereby decreasing any confounding factors during the implementation of the new protocol. All neonates’ data were gathered from the duration of their inpatient stay. Neonates were screened and graded by the same two pediatric ophthalmologists (K. H and C. B) throughout the study.

### Eligibility

Eligibility for inclusion in these data was classified as neonates discharged from the Riley Hospital for Children NICU between January 2017 and December 2022 who had a birthweight < = 1500 g, were < = 30 weeks gestation at birth, had no congenital abnormalities, and had Zone 1 Stage 2 w/o plus or Zone 2 Stage 2 w/o plus ROP. Preterm infants with type 1 ROP were immediately treated with laser photocoagulation or intravitreal bevacizumab [18]. 

### Clinical data

All data were collected by the authors in compliance with the Indiana University School of Medicine IRB and HIPAA. Demographic data and clinical variables known to affect ROP (sex, race, birthweight, gestational age, diagnosis of necrotizing enterocolitis (NEC), interventricular hemorrhage (IVH), sepsis, bronchopulmonary dysplasia (BPD), and admission respiratory support) were retrieved from electronic records. The primary endpoint of this study was the need for invasive intervention via laser photocoagulation or intravitreal bevacizumab. Secondary endpoints were the duration of hospital stay and time on respiratory support.

### Oxygen supplementation protocol

The Riley Hospital oxygen protocol was originally conceived based on the research of the STOP-ROP trial. Prior to protocol implementation, all preterm infants were treated with an oxygen saturation target of 91–95% during the duration of their hospitalization. After implementation, all neonates diagnosed with stage 2 ROP had a new target oxygen saturation of 97–99% (Fig. 1). Any adjustments in oxygen support modality or changes in FiO2 were managed by the bedside nurse. Given the potential risks of hyperoxia in neonates with chronic lung disease, neonates’ FiO2 was titrated based on their level of respiratory support. For infants requiring invasive mechanical ventilation (high-frequency ventilators (HFV), conventional mechanical ventilators (CMV) or any noninvasive mechanical ventilation providing continuous positive airway pressure (CPAP), a limit of 50% maximum FiO2 was allowed to avoid oxygen toxicity. Infants on ≤ 1 L per minute nasal cannula (NC) were placed on 100% FiO2. The decision to continue supplemental oxygen therapy was reassessed following weekly ophthalmology exams after initiation of oxygen treatment.

### Statistical analysis

Descriptive statistics were obtained for demographic values at baseline for each cohort. To explore the differences between the two cohorts, chi-square tests were performed for categorical variables, and t tests were performed for continuous variables, which were first explored for normality. The main outcome of patients needing laser or bevacizumab was first explored unadjusted with chi-squared tests and later analyzed using a logistic regression adjusting for clinically or statistically significant covariates (sex, race, birthweight, gestational age, NEC status, IVH grade, history of sepsis, BPD, admission respiratory support need, and days on each respiratory modality). The outcomes, length of stay, time on CPAP, time on NC and time on any respiratory support, were analyzed with a linear regression adjusting for the same variables as the main outcomes. Assumptions for normality were checked and met. All data were analyzed using R v4.3.1 and RStudio v2023.09.1 + 494 software [19]. 

## Results

### Baseline demographics

A total of 212 patients met the criteria for inclusion, with 122 patients in Cohort A, preprotocol implementation, and 89 patients in Cohort B, postprotocol implementation. All demographic data are summarized in Table 1. Overall, there was no difference in the distribution of sex, Caucasian vs. non-Caucasian status, birthweight, diagnosis of NEC, BPD, or IVH, or sepsis. Patients from Cohort B had marginally higher gestational age at birth (Cohort A 25.3 ± 1.90, Cohort B 25.8 ± 1.84, *p* = 0.04), and a lower CMV use with higher CPAP use for respiratory support during admission (Table 1). We next assessed the days of respiratory support as a proxy for pulmonary health for both cohorts and compared the days on each respiratory support modality (Table 2). We found that Cohort A had a longer duration on HFV and CMV, and Cohort B had a longer duration on CPAP. Overall, Cohort B had lower days on any respiratory support. Given the statistical significance, we included days of ventilation as a covariate in subsequent logistic regression.

**Table 1:** Summary of the demographic data between Cohort A and Cohort B. Only admission respiratory support was significantly significant between the two cohorts. Chi-square tests were performed for categorical variables, and t-tests were performed for continuous variables. NEC-necrotizing enterocolitis, IVH – intraventricular hemorrhage, RA – room air, NC – nasal cannula, CPAP – continuous positive airway pressure, CMV- conventional mechanical ventilation, HFV – high-frequency ventilation

	Cohort A (*N* = 122)	Cohort B (*N* = 89)	Overall (*N* = 211)	*P*-value
**Sex**				
F	57 (46.7%)	42 (47.2%)	99 (46.9%)	1.00
M	65 (53.3%)	47 (52.8%)	112 (53.1%)	
**Caucasian**				
N	57 (46.7%)	47 (52.8%)	104 (49.3%)	0.46
Y	65 (53.3%)	42 (47.2%)	107 (50.7%)	
**Birthweight (gram)**				
Mean (SD)	717 (206)	763 (219)	737 (212)	0.12
Median [Min, Max]	680 [350, 1380]	7.40 [410, 1450]	690 [350, 1450]	
**Gestational Age (week)**				
Mean (SD)	25.3 (1.90)	25.8 (1.84)	25.5 (1.88)	0.04
Median [Min, Max]	25.0 [22.4, 36.6]	25.4 [22.7, 32.6]	25.1 [22.4, 36.6]	
**NEC**				
No	89 (73.0%)	68 (76.4%)	157 (74.4%)	0.68
Yes	33 (27.0%)	21 (23.6%)	54 (25.6%)	
**IVH (Grade)**				
No IVH	69 (56.6%)	49 (55.1%)	118 (55.9%)	0.06
Mild IVH	26 (21.3%)	10 (11.2%)	36 (17.1%)	
Severe IVH	27 (22.1%)	30 (33.7%)	57 (27.0%)	
**Sepsis**				
No	75 (61.5%)	64 (71.9%)	139 (65.9%)	0.15
Yes	47 (38.5%)	25 (28.1%)	72 (34.1%)	
**BPD**				
No	5 (4.1%)	7 (7.9%)	12 (5.7%)	0.387
Yes	117 (95.9%)	82 (92.1%)	199 (94.3%)	
**Admit resp support**				
CMV	68 (55.7%)	35 (39.3%)	103 (48.8%)	
CPAP	9 (7.4%)	22 (24.7%)	31 (14.7%)	
HFV	43 (35.2%)	31 (34.8%)	74 (35.1%)	0.004
NC	1 (0.8%)	1 (1.1%)	2 (0.9%)	
RA	1 (0.8%)	0 (0%)	1 (0.5%)	
**Survived to DC**				
No	5 (4.1%)	2 (2.2%)	7 (3.3%)	0.701
Yes	117 (95.9%)	87 (97.8%)	204 (96.7%)	

**Table 2:** Days on respiratory support. Cohort A had more days spent on HFV and CMV, while Cohort B had a longer duration on CPAP. Analysis was performed using chi-square test

	Cohort A (*N* = 122)	Cohort B (*N* = 89)	Overall (*N* = 211)	*P*-value		
Days on **HFV**						
Mean (SD)	23.0 (22.6)	16.5 (17.3)	20.2 (20.8)	0.02		
Median [Min, Max]	19.5 [0, 89.0]	13.0 [0, 64.0]	15.0 [0, 89.0]			
**CMV**						
Mean (SD)	86.8 (74.8)	45.9 (56.1)	69.5 (70.4)	< 0.001		
Median [Min, Max]	68.5 [0, 299]	23.0 [0, 226]	45.0 [0, 299]			
**CPAP**						
Mean (SD)	41.3 (32.4)	68.4 (39.2)	52.8 (37.8)	< 0.001		
Median [Min, Max]	42.5 [0, 117]	69.0 [0, 268]	56.0 [0, 268]			
**NC**						
Mean (SD)	16.7 (49.6)	17.4 (16.3)	17.0 (39.1)	0.876		
Median [Min, Max]	7.00 [0, 402]	13.0 [0, 70.0]	11.0 [0, 402]			
**Any resp support**						
Mean (SD)	168 (79.7)	149 (66.3)	160 (74.7)	0.0626		
Median [Min, Max]	161 [26.0, 526]	130 [27.0, 493]	149 [26.0, 526]			

### Implementation of the oxygen supplemental protocol is associated with a decreased need for laser photocoagulation or intravitreal bevacizumab

Overall, we found a significant reduction in the need for subsequent laser and/or intravitreal bevacizumab treatment in infants who developed stage 2 ROP after the implementation of supplemental O2 therapy (Table 3). In Cohort A, 55% (67/122) of infants with stage 2 ROP needed treatment, versus only 22% (20/89) of infants in Cohort B who needed further laser or intravitreal bevacizumab treatment (*P* < 0.001, univariate Pearson’s Chi-squared test). We next performed a logistic regression model adjusting for clinically important and significantly different covariates (sex, race, birthweight, gestational age, NEC status, IVH grade, sepsis, BPD, admission respiratory support need, and days on each respiratory modality). Compared to Cohort A, patients in Cohort B had a significantly decreased risk of needing laser and/or intravitreal bevacizumab treatment. (Table 4, OR = 0.19; 95% CI 0.09–0.41; *P* = 0.000). We also found that female sex, higher birthweight, sepsis, and longer time on conventional ventilation were factors associated with decreased need for laser or intravitreal bevacizumab. NEC was associated with higher odds of needing laser and/or intravitreal bevacizumab.

**Table 3:** Pearson’s chi-square tests comparing the need for laser photocoagulation and/or IV bevacizumab in Cohort A vs. Cohort B

Laser Photocoagulation/Intravitreal Bevacizumab	Cohort A	Cohort B	Total	*p*-value ^1^
Yes	67 (55%)	20 (22%)	87 (41%)	
No	55 (45%)	69 (78%)	125 (59%)	< 0.001
**Total**	122 (100%)	89 (100%)	211 (100%)	

^1^ Pearson’s Chi-squared test

**Table 4:** Logistic regression analysis of factors associated with the odds ratio (OR) of needing laser photocoagulation and IV bevacizumab. The following covariates were included in the logistic regression (sex, Caucasian status, birthweight, gestational age, NEC, IVH, sepsis, BPD, admit respiratory support, days on different types of respiratory support)

Factors	Odds Ratio	95% CI	*p*-value
Supplemental O2			
No - Cohort A	(reference)		
Yes - Cohort B	0.19	0.08–0.40	0.000
Sex			
Female		(reference)	
Male	2.18	1.10–4.46	0.028
Caucasian	1.05	0.53–2.06	0.895
Birthweight (per 100 gram)	0.67	0.53–0.84	0.001
Gestational Age (week)	0.89	0.70–1.11	0.310
NEC	2.30	1.05–5.19	0.040
IVH No IVH Mild (Grade 1, 2) Severe (Grade 3,4)	2.63 1.26	(reference) 1.02–7.06 0.56–2.88	0.048 0.570
Sepsis	0.40	0.18–0.82	0.016
BPD	0.42	0.09–2.16	0.276
Days on HFV	1.02	1.00–1.04	0.088
Days on CMV	0.99	0.99–1.00	0.043
Days on CPAP	1.00	0.98–1.01	0.546
Days on NC	1.00	0.98–1.00	0.409

### O2 protocol is associated with longer NC/O2 use but does not increase LOS

Finally, we assessed whether implementation of the supplemental O2 protocol in our population impacted the time on oxygen and length of stay in the hospital. After correcting for the same variables as the main treatment outcome, time on CPAP was increased (Beta = 18 [8.7,27], *P* = 0.000). However, the length of stay did not differ between the two cohorts (Beta = 12 [-0.58,24], *P* = 0.062)

## Discussion

The survival of premature infants has steadily increased over the past few decades [20]. However, ROP remains a significant disease affecting surviving preterm infants born ≤ 30 weeks GA. Specifically, over 50% of these infants had a diagnosis of ROP, with 1 in 10 preterm infants developing severe (Stage 3) ROP [20]. Currently, the prevention of severe ROP development remains limited. Limiting oxygen exposure by targeting lower oxygen saturations (85–89%) during the vaso-obliterative phase is the only management shown to decrease the rate of severe ROP but is associated with a higher mortality rate in preterm infants [2, 12, 13, 15]. Other therapies during the early postnatal NICU course, such as aggressive parenteral nutrition, use of human milk, and several vitamin supplements, have been shown to decrease the risks of ROP of all stages, but the results on severe ROP needing intervention were mixed [21]. The aim of this study was to assess whether the use of supplemental oxygen to achieve a higher oxygen saturation target after the onset of the vaso-proliferative phase in preterm infants with ROP will dampen the conversion to Type 1 ROP and result in a lower rate of laser photocoagulation or intravitreal bevacizumab. Indeed, we found that after implementing the supplemental oxygen protocol, infants with Zone 1 or Zone 2 Stage 2 ROP without plus disease in Cohort B had lower rates of disease progression needing laser photocoagulation or intravitreal bevacizumab. This finding is consistent with benefits reported in a meta-analysis (2 RCTs, 1 prospective observation, and 2 retrospective cohorts with over 1600 patients) [22] as well as a retrospective cohort study by Colaizy et al. [16] Collectively, these studies found that higher oxygen saturation targets after 32 weeks of CGA are associated with decreased ROP disease progression.

We also assessed the duration of respiratory support and length of stay (LOS) in our patients to assess whether implementation of the supplemental oxygen protocol resulted in a longer duration of hospital stay or a longer duration of need for respiratory support. We found that Cohort B had lower number of days on HFV and CMV, higher number of days on CPAP, and lower number of days on any respiratory support with no increase in LOS. The difference in days on invasive mechanical ventilation and higher days on CPAP likely reflected changes in respiratory management (more proactive use of CPAP and extubation attempts), and the implementation of the protocol did not result in prolonged use of oxygen or LOS. Survival to discharge was not different in infants who were placed on supplemental oxygen protocol. These data also suggest that limiting FiO2 in high-risk infants to 50%, as we did in our protocol, does not increase adverse pulmonary events, as reported in the STOP-ROP trial. Nevertheless, our study is limited by the nature of its retrospective cohort design and therefore unable to control for nonsystematic changes in clinical practice. However, major clinical management (e.g., surfactant delivery, nutritional management) remained consistent. Furthermore, we had two pediatric ophthalmologists who had consistent ROP grading and management.

## Conclusions

In conclusion, our results showed that the use of supplemental oxygen to target higher oxygen saturations in infants with Zone 1 and Zone 2 Stage 2 ROP without plus disease is safe and potentially decreases disease progression and the need for treatment with laser photocoagulation or intravitreal bevacizumab.

## Data Availability

The datasets used and/or analyzed during the current study are available from the corresponding author on reasonable request.

## Abbreviations

ROP: Retinopathy of Prematurity
NEC: necrotizing enterocolitis
IV: intravitreal
CGA: corrected gestational age
IVH: intraventricular hemorrhage
RA: room air
NC: nasal cannula
CPAP: continuous positive airway pressure
CMV: conventional mechanical ventilation
HFV: high–frequency ventilation
